# Potential mechanism of Qigebikang tablets in the treatment of allergic rhinitis: Network pharmacology and molecular docking

**DOI:** 10.1097/MD.0000000000044321

**Published:** 2025-09-19

**Authors:** Shan Liu, Jinglan Lin, Lifen Chen, Zhihao Huang, Yanwen Cai

**Affiliations:** a Department of Otolaryngology, Dongguan Hospital of Guangzhou University of Chinese Medicine, Dongguan, Guangzhou, China.

**Keywords:** allergic rhinitis, molecular docking, network pharmacology, Qige Bikang tablets

## Abstract

This study employed network pharmacology and molecular docking to investigate Qige Bi Kang tablets’ (QGBK) mechanism against allergic rhinitis (AR). Active ingredients and targets of QGBK were sourced from traditional Chinese medicine systems pharmacology database. AR-related targets were obtained from GeneCards and TTD. The intersection targets were analyzed in STRING for protein–protein interactions, followed by topological analysis in Cytoscape to identify core targets. The R package clusterProfiler performed Gene Ontology and Kyoto Encyclopedia of Genes and Genomes enrichment analysis. AutoDock validated molecular docking between key QGBK ingredients and critical targets. We identified 1174 QGBK active ingredients and 453 AR-related targets. Topological analysis of the protein–protein interaction network yielded 10 core targets. The top 5 targets by degree were signal transducer and activator of transcription (STAT)3, interleukin (IL-6), glyceraldehyde-3-phosphate dehydrogenase, tumor necrosis factor, and nuclear factor-k-gene binding 1. The top 4 key active ingredients by degree were Quercetin, Kaempferol, Emodin, and Palmitic acid. Molecular docking showed binding energies < −3.783 kcal/mol between these molecules and targets, indicating strong affinity. Gene Ontology enrichment revealed QGBK’s AR treatment primarily involves responses to polysaccharides, bacterial molecules, xenobiotic stimuli, and positive MAPK cascade regulation. Kyoto Encyclopedia of Genes and Genomes analysis highlighted the AGE-RAGE and IL-17 signaling pathways as significant. In conclusion, QGBK’s anti-AR mechanism exhibits multicomponent, multi-target, multi-pathway regulation. Quercetin, Kaempferol, Emodin, and Palmitic acid are key therapeutic components, acting on core targets like STAT3, IL-6, glyceraldehyde-3-phosphate dehydrogenase, and MMP9. QGBK exerts its effects through triple mechanisms: anti-inflammation, immunomodulation, and mitigation of airway responsiveness, primarily via STAT3/IL-6 signaling modulation. However, further in vivo and clinical validation is essential. Future research should prioritize unlocking QGBK’s clinical translation potential and advancing targeted formulation development.

## 1. Introduction

Allergic rhinitis (AR) is a chronic inflammatory disease of the nasal mucosa characterized by nasal itching, sneezing, rhinorrhea, and nasal congestion. It is mediated by allergen-induced immunoglobulin E (IgE).^[1]^ Despite seemingly simple symptoms, it significantly impairs patients’ quality of life. Studies in Europe and America indicate that 20% to 30% of adults and up to 40% of children suffer from this condition,^[2]^ while its prevalence in China has been rising annually, currently reaching 38%.^[3]^

Current clinical treatments (such as antihistamines, glucocorticoids, and immunotherapy) often face limitations including significant side effects (drowsiness, drug-induced rhinitis, etc) and suboptimal efficacy.^[4,5]^ Traditional Chinese medicine (TCM), recognized for its high safety profile and multi-target effects, has emerged as a potential alternative therapy.^[6,7]^ The Chinese Guidelines for the Diagnosis and Treatment of Allergic Rhinitis (2022 Revised Edition) explicitly incorporates TCM into AR treatment protocols.^[8]^ Although classical TCM texts do not directly mention AR, descriptions of conditions like “Bi Qiu” (nasal blockage with clear discharge) closely align with AR. Retrospective studies show that 163 Chinese herbs have been used historically to treat AR-like symptoms, most of which remain in clinical use today.^[9]^

Qige Bi Kang tablets (QGBK, Guangdong Pharmaceutical Approval No. Z20110001) are a hospital preparation widely used at Dongguan Hospital of Traditional Chinese Medicine for treating AR with significant efficacy. Composed of Hedysarum multijugum Maxim (HMM), Saposhnikoviae Radix (SR), Paeoniae Radix Alba (PPA), Cicadae Periostracum (CP), Radix Puerariae (RP), Xanthii Fructus (XF), Polygonati Odorati Rhizoma (POR), *Beauveria bassiana* Vaillant (BBV), Atractylodes macrocephala Koidz (AMK), Cimicifuga Rhizoma (CR), Tribulifructus (T), licorice (L), it possesses immunomodulatory and anti-inflammatory properties. A 2017 study found that the modified Yupingfeng Formula (YPF, primarily containing HMM, SR, AMK) with added Xanthii Fructus suppressed eosinophil infiltration and mast cell degranulation, improved nasal mucosal remodeling, and thereby alleviated AR symptoms.^[10]^ Subsequent experiments confirmed that this formula inhibits inflammatory responses by regulating the Th1/Th2 balance.^[11]^ QGBK incorporates insect-derived medicines like CP and Bombyx Batryticatus into the Yupingfeng San base. Component studies indicate: CP extract inhibits mast cell degranulation,^[12–14]^ and Polygonati Odorati Rhizoma polysaccharides exhibit immunomodulatory functions.^[15,16]^ However, the specific molecular mechanisms of QGBK in treating AR remain unclear, and the associations between its active components and disease targets, as well as its signaling pathways, require further exploration.

Network pharmacology is an interdisciplinary field that integrates informatics and biology. Through multi-angle and multi-level network analysis, it enables the elucidation of signaling pathways and molecular targets involved in drug treatment of diseases, facilitating the study of the mechanisms of action of TCM formulas and the screening of active components. This study integrates network pharmacology with molecular docking technology, focusing on the core active ingredients, therapeutic targets, key pathways, and underlying network pharmacology mechanisms of QGBK in treating AR.

Not only has this study pioneered the construction of a multidimensional regulatory network of “QGBK-active ingredients-core targets-signaling pathways,” but the established methodological framework for TCM compound research further provides a paradigm reference for elucidating the mechanisms of similar formulations. The research outcomes demonstrate demonstrable application prospects in fields including novel drug development for respiratory immune diseases and modernization of classic TCM formulas.

## 2. Materials and methods

### 2.1. Acquisition of QGBK active ingredient targets

Based on the Traditional Chinese Medicine Systems Pharmacology Database (traditional Chinese medicine systems pharmacology database; https://www.tcmsp-e.com/index.php), the chemical constituents of QGBK’s component herbs (Astragali Radix/HMM, Saposhnikoviae Radix/SR, Paeoniae Radix Alba/PRA, Cicadae Periostracum/CP, Puerariae Lobatae Radix/RP, Xanthii Fructus/XF, Polygonati Odorati Rhizoma/POR, Bombyx Batryticatus/BBV, Atractylodis Macrocephalae Rhizoma/AMK, Cimicifugae Rhizoma/CR, Tribuli Fructus/T, Glycyrrhizae Radix et Rhizoma/L) were retrieved. Screening criteria: oral bioavailability (OB) ≥ 30% and drug-likeness (DL) ≥ 0.18. After removing duplicate components, candidate active ingredients and their corresponding targets were obtained.

### 2.2. Screening of AR disease targets

Using “Hypersensitive rhinitis” and “Allergic rhinitis” as keywords, AR-related genes were retrieved from the GeneCards (https://www.genecards.org/) and TTD (https://db.idrblab.net/ttd/) databases. Search results were merged, duplicate genes were removed, and the AR disease target set was compiled. GeneCards: A searchable gene database integrating resources from ~150 gene-centric databases, providing information on nearly all known human genes. TTD: The world’s first open-access online database offering global drug target information.

### 2.3. Venn diagram analysis

The Venny 2.1 online tool (https://jvenn.toulouse.inra.fr/app/index.html) was used to generate a Venn diagram. The intersection of QGBK active ingredient targets and AR disease targets yielded potential therapeutic targets.

### 2.4. PPI network construction and core target screening

Common targets were imported into the STRING database (https://string-db.org/), with species set to Homo sapiens, to construct a protein–protein interaction (PPI) network. The top 10 core targets were screened using the Maximal Clique Centrality algorithm in Cytoscape, generating a simplified PPI network. Cytoscape 3.8.2 was employed to build a 4-dimensional network model, where core active ingredients were identified based on topological parameters (degree, betweenness centrality, closeness centrality) to reconstruct the PPI network.

### 2.5. GO and KEGG pathway enrichment analysis

The R package clusterProfiler was used for Gene Ontology (GO) and Kyoto Encyclopedia of Genes and Genomes (KEGG) pathway enrichment analyses. Screening criteria: adjusted *P*-value < .05. The top 10 GO terms (Biological Process/BP, Molecular Function/MF, Cellular Component/CC) and top 20 KEGG pathways were visualized.

### 2.6. Molecular docking

To elucidate QGBK’s therapeutic mechanism in AR treatment, molecular docking was performed on core targets identified by intersecting 12 drugs and disease targets, as well as top-ranked targets from docking studies. The top 10 relevant targets (ranked by Maximal Clique Centrality in the PPI network) and top 10 molecules were selected. 3D structure files (sdf format) of the 10 compounds were downloaded from PubChem. Receptor PDB files for key targets were obtained from the RCSB database (https://www.rcsb.org). Ligand and receptor data were imported into AutoDock for molecular docking, and binding energies were recorded. AutoDock: An open-source molecular simulation software primarily used for docking small molecules to biomacromolecules. Visualization: Docking results were visualized using PyMOL.

## 3. Results

### 3.1. Common targets of QGBK and AR

**Figure 1.**
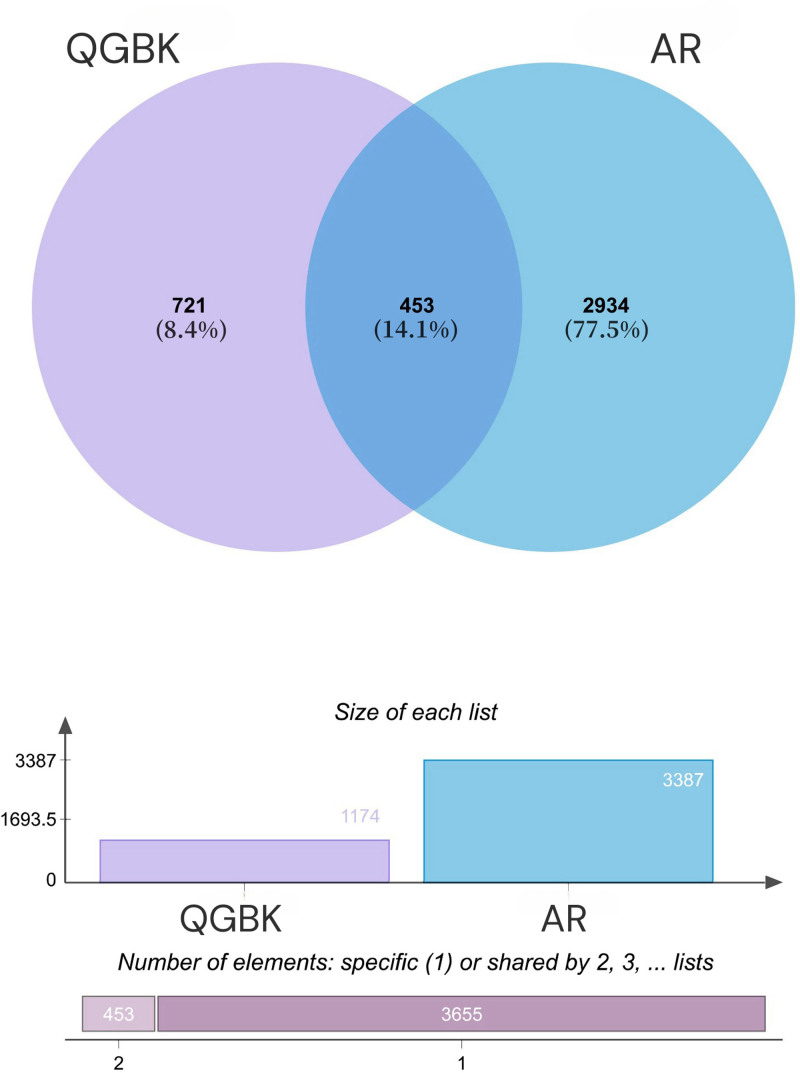
Venn diagram of interaction targets between QGBK and AR. AR = allergic rhinitis, QGBK = Qige Bi Kang.

Using the Venny 2.1 online tool, the intersection of 1174 QGBK targets and 3387 AR targets yielded 453 overlapping targets (Fig. 1).

### 3.2. Construction of the “Herb-Ingredient-Target-Disease” network

**Figure 2.**
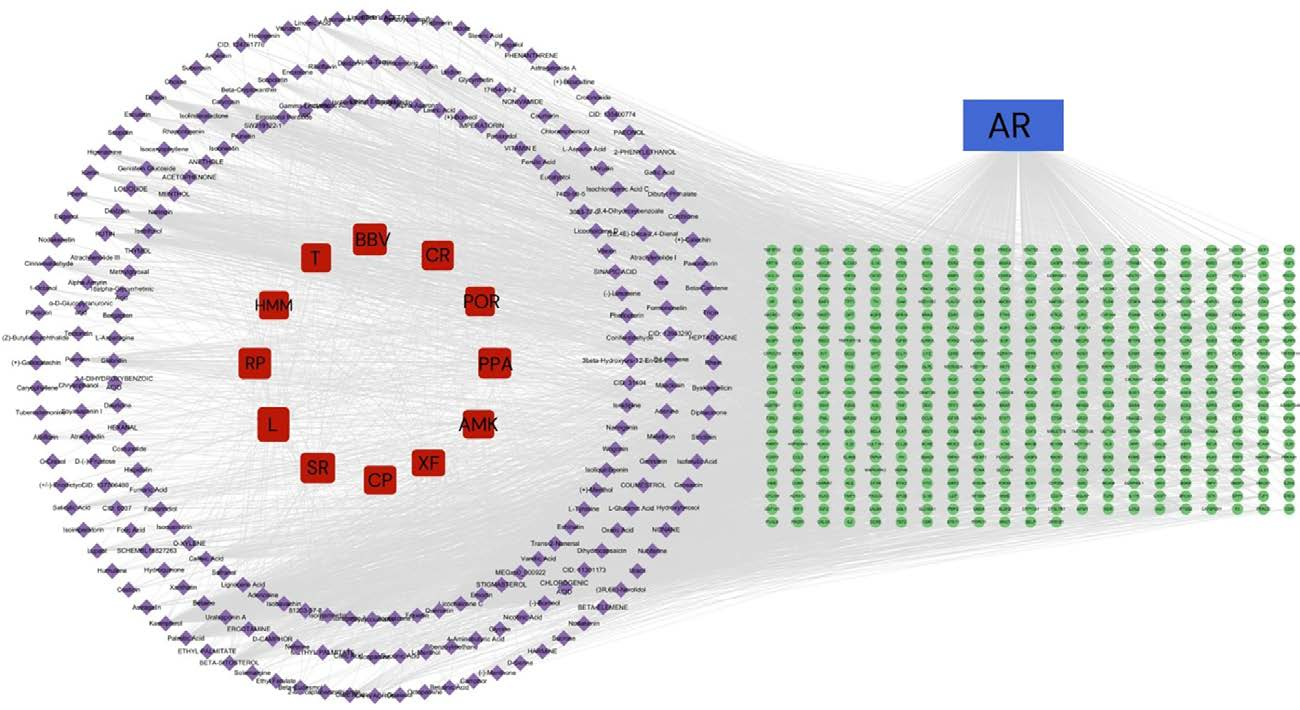
“Herb-Ingredient-Target” network. Red nodes: herbs; purple nodes: ingredients; green nodes: potential gene targets.

Active ingredients of QGBK and the overlapping targets were imported into Cytoscape 3.8.2 to construct a “Herb-Ingredient-Target” network (Fig. 2). Topological analysis identified the top 5 key active ingredients by degree value (Table 1).

**Table 1:** Key active ingredients of QGBK for AR treatment.

Molecule name	Degree	Betweenness centrality	Closeness centrality
Quercetin	516	0.054481328	0.424984691
Kaempferol	215	0.011124649	0.374932469
Emodin	114	0.012465733	0.376559957
Palmitic acid	96	0.005784843	0.367779544
Daidzein	90	0.00750743	0.360145304
Genistein	78	0.016584706	0.37737901
α-Sitosterol	72	0.005439507	0.374932469
Rutin	72	0.003859999	0.356445814
Dioscin	70	0.014512465	0.376151762
Paeoniflorin	62	0.005118097	0.358286009

AR = allergic rhinitis, QGBK = Qige Bi Kang.

### 3.3. Core target prediction and PPI network analysis

**Figure 3.**
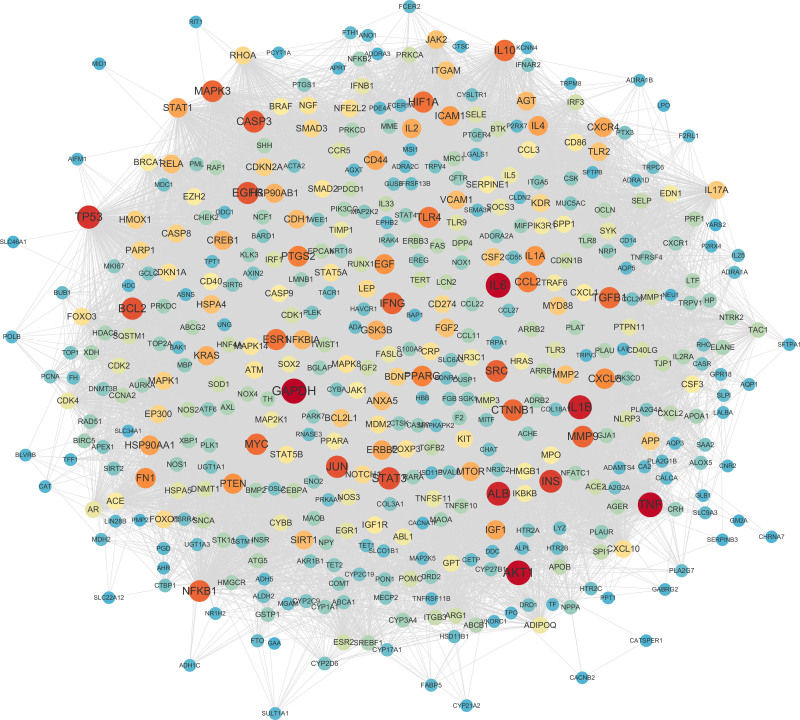
PPI network of QGBK for AR treatment. PPI = protein–protein interaction, QGBK = Qige Bi Kang.

**Figure 4.**
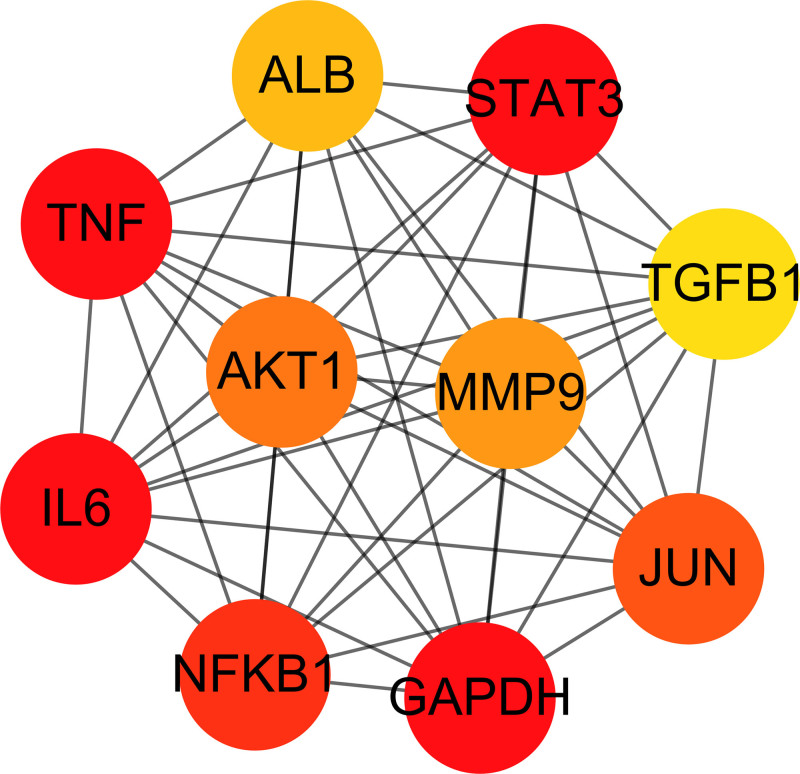
PPI network of core targets. Redder and larger nodes indicate higher interaction degrees. PPI = protein–protein interaction.

The 453 overlapping targets were imported into the STRING database to generate a PPI network (Fig. 3). Results were downloaded and imported into Cytoscape 3.8.2, where 10 core targets were screened based on the average values of degree, closeness, and betweenness centrality. A core target PPI diagram was generated according to degree values (Fig. 4). Signal transducer and activator of transcription (STAT)3, interleukin (IL)-6, glyceraldehyde-3-phosphate dehydrogenase (GAPDH), tumor necrosis factor, nuclear factor-k-gene binding 1, JUN, AKT1, MMP9, ALB, and TGFB1 were identified as key targets for QGBK in treating AR.

### 3.4. Functional enrichment and pathway network analysis

#### 3.4.1. GO enrichment analysis

**Figure 5.**
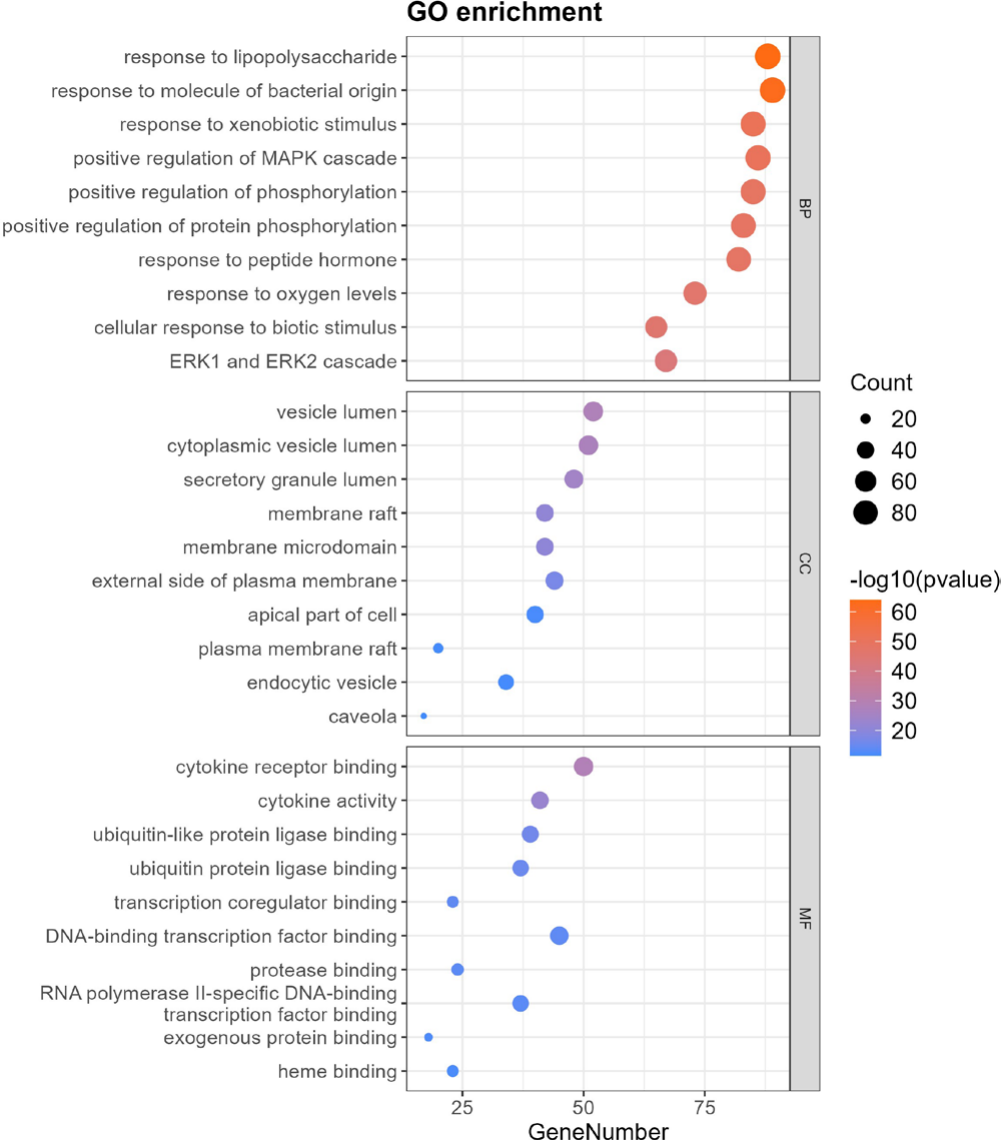
Top 10 GO enrichment results for BP, CC, and MF. BP = biological process, CC = cellular component, GO = Gene Ontology, MF = molecular function.

Top 10 entries for Biological Process (BP), Cellular Component (CC), and Molecular Function (MF) were visualized in bubble plots (Fig. 5): BP: Responses to lipopolysaccharide, bacterial molecules, xenobiotic stimuli; positive regulation of MAPK cascade, phosphorylation. CC: Vesicle lumen, cytoplasmic vesicle lumen, secretory granule lumen, membrane raft, membrane microdomain, external side of plasma membrane, cell apex, plasma membrane raft. MF: Cytokine receptor binding, cytokine activity, ubiquitin-like protein ligase binding, ubiquitin-protein ligase binding, transcription coregulator binding, DNA-binding transcription factor binding.

#### 3.4.2. KEGG pathway analysis

**Figure 6.**
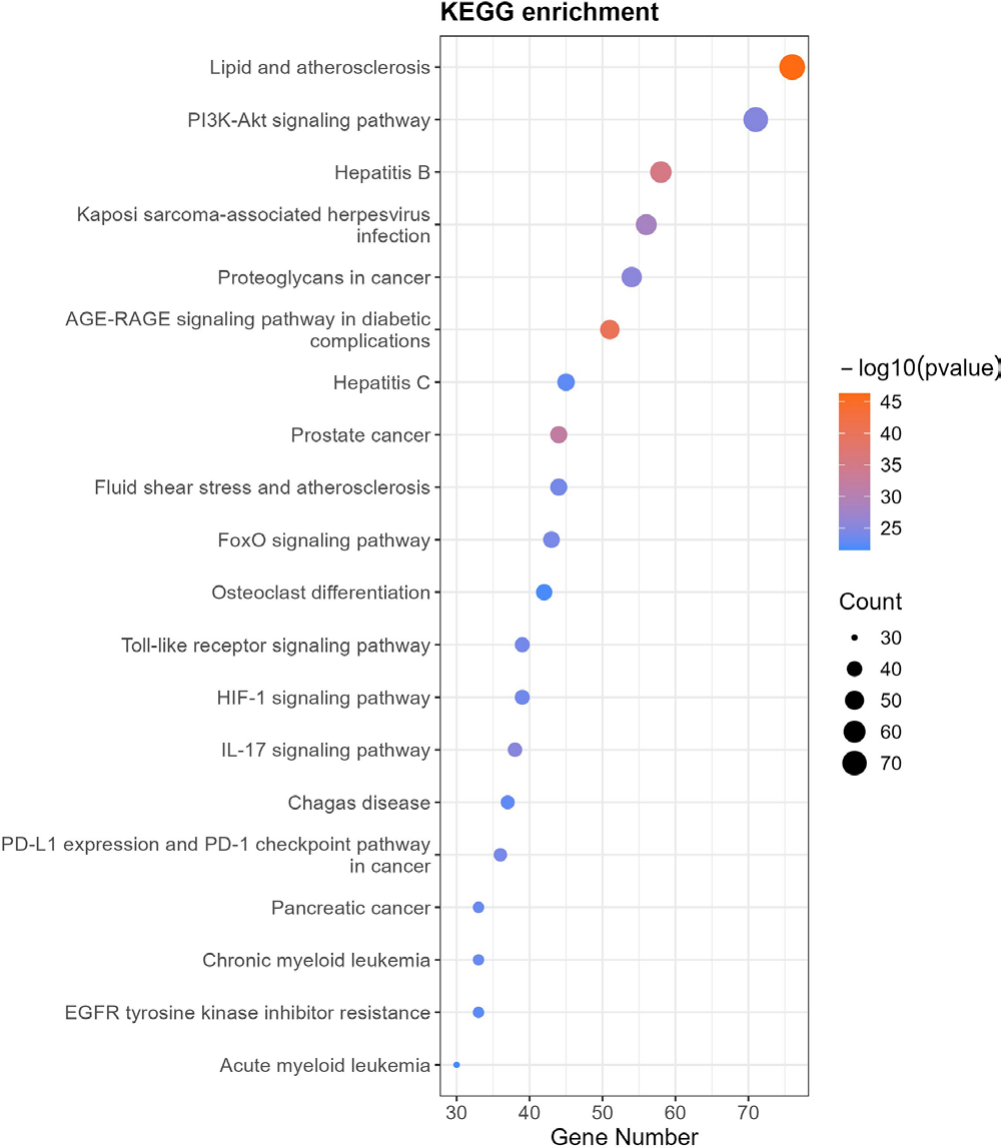
Horizontal bar chart of top 20 KEGG pathways. KEGG = Kyoto Encyclopedia of Genes and Genomes.

Top 20 KEGG pathways were enriched in a horizontal bar chart (Fig. 6), including:

Lipid and atherosclerosis, AGE-RAGE signaling pathway in diabetic complications, IL-17 signaling pathway, PI3K-Akt signaling pathway, FoxO signaling pathway, Toll-like receptor signaling pathway, HIF-1 signaling pathway, EGFR tyrosine kinase inhibitor resistance, human cytomegalovirus infection, prolactin signaling pathway, C-type lectin receptor signaling pathway, Chemical carcinogenesis – receptor activation, Th17 cell differentiation, MAPK signaling pathway.

### 3.5. Molecular docking validation

**Figure 7.**
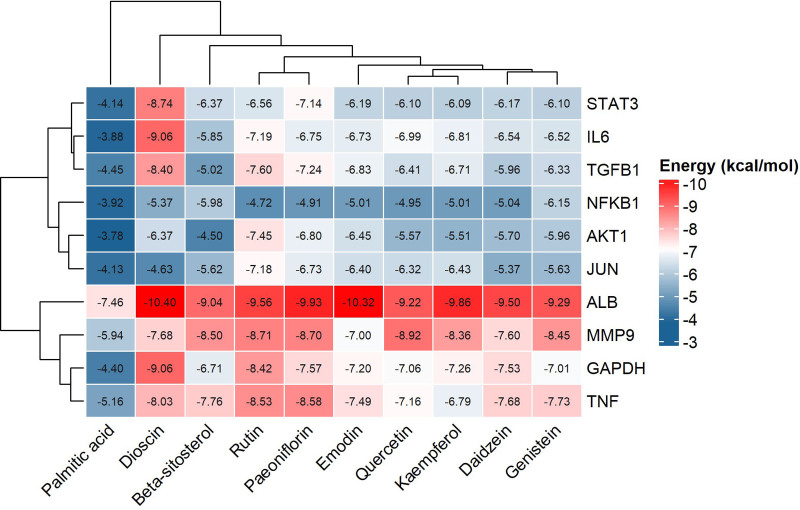
Heatmap of molecular docking energy scores (kcal/mol) for key components and targets. Lower scores are shown in redder colors.

**Figure 8.**
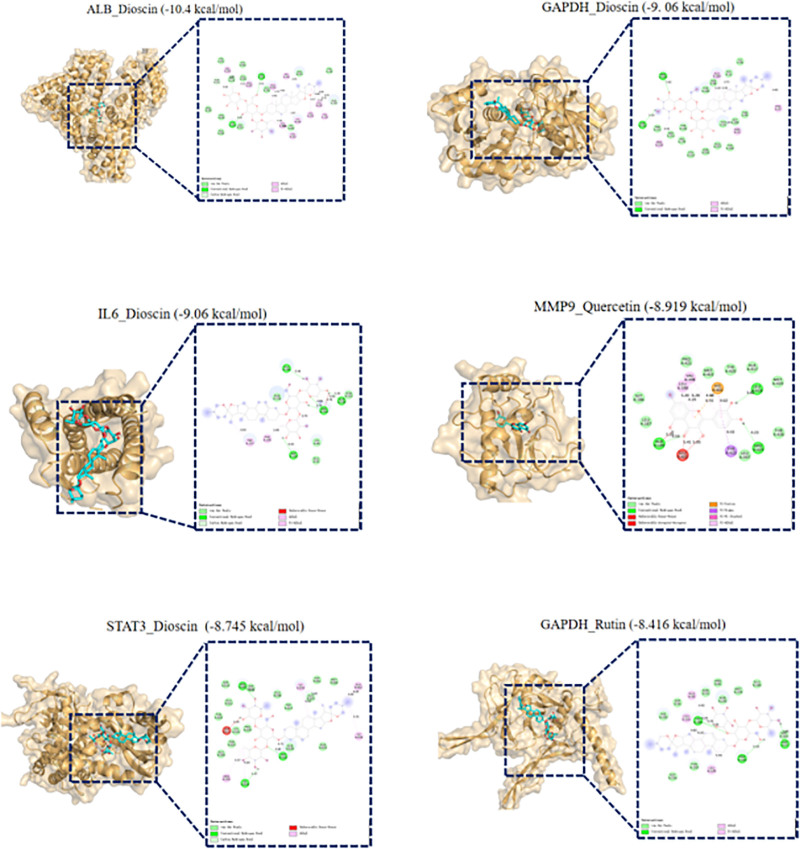
Schematic diagrams of molecular docking modes.

Molecular docking was performed between key active components (Table 1) and the top 5 core target proteins by degree value. All docking energies were <−3.783 kcal/mol, indicating strong binding affinity. Docking scores are displayed in a heatmap (Fig. 7). Results confirmed stable binding between QGBK’s key components (Quercetin, Kaempferol, Emodin, Palmitic acid) and core targets (ALB, GAPDH, IL-6, MMP9, STAT3), visualized in Figure 8.

## 4. Discussion

In the United States and Europe, over 40% of the population is at risk of allergies, with the frequency of sensitization to inhaled allergens on the rise. AR is triggered by common allergens including seasonal pollen and molds, as well as perennial indoor allergens such as dust mites, pets, pests, and certain fungi.^[17]^ Currently, the most commonly prescribed medications include mast cell stabilizers (sodium cromoglicate), intranasal corticosteroids (fluticasone propionate), antihistamines (loratadine), decongestants (oxymetazoline hydrochloride), and leukotriene receptor antagonists (montelukast sodium).^[18]^ However, long-term use of these drugs in AR patients may lead to adverse effects, such as rhinorrhea induced by intranasal corticosteroids,^[19]^ drowsiness caused by antihistamines,^[20]^ and rhinitis medicamentosa resulting from decongestant overuse.^[21]^ Due to its demonstrated safety and efficacy, TCM is increasingly utilized for AR management.^[22]^

Approved by the Guangdong Provincial Medical Products Administration, Qige Bi Kang tablets (QGBK) represent a patented TCM formulation derived from herbal compounds. While its efficacy against AR has been scientifically validated, the precise mechanism remains unclear. Network pharmacology can elucidate drug mechanisms in diseases by establishing molecular interaction networks and signal transduction models.^[23,24]^ This study employed network pharmacology to explore QGBK’s therapeutic targets and mechanisms in AR.

Thus, our research team utilized network pharmacology and molecular docking to investigate QGBK’s core components, primary targets, and potential therapeutic pathways for AR treatment. Our findings revealed that components including Quercetin, Kaempferol, Emodin, and Palmitic acid occupied central positions in the network, indicating their roles as key active constituents responsible for the formula’s therapeutic effects. Molecular docking confirmed strong binding affinity between these components and targets, with STAT3, IL-6, GAPDH, and MMP9 demonstrating optimal binding to multiple compounds.

Quercetin exhibits anti-allergic functions by inhibiting histamine production and pro-inflammatory mediators. It plays a primary role in anti-inflammatory and immunomodulatory processes, regulates Th1/Th2 stability, and reduces antigen-specific IgE antibody release from B cells^.[25]^ Experimentally, quercetin decreased sneezing frequency in mice and significantly suppressed increases in substance P, calcitonin gene-related peptide, and nerve growth factor in nasal lavage fluid.^[26]^ Kaempferol has been shown to reduce eosinophil accumulation in airways and lung tissues^[27]^ and attenuates Lyn kinase activation to prevent mast cell-mediated allergic diseases.^[28]^ Yue Lu et al^[29]^demonstrated in murine studies that Emodin inhibited degranulation, eicosanoid production, and cytokine secretion (tumor necrosis factor-α and IL-6) in IgE/Ag-stimulated mast cells in a dose-dependent manner. Orally administered Emodin also alleviated mast cell-dependent passive anaphylaxis in IgE-sensitized mice. Palmitic acid serves as a precursor for sphingosine-1-phosphate (S1P), a dynamic lipid mediator whose signaling is implicated in allergen-induced eosinophilic inflammation, airway hyperreactivity, and immune cell trafficking.^[30]^ Recent animal research^[31]^ highlights the potential role of palmitic acid composition and lipid balance in the pathophysiology of allergic airway inflammation.

STAT3, a transcription factor activated by cytokines (e.g., IFN, IL-2, IL-6, IL-10, IL-22, EGF), regulates innate and adaptive immune responses.^[32]^ Simeone et al found that STAT3 activation reduces airway hyperreactivity and significantly mitigates house dust mite-induced pulmonary inflammation.^[33]^ IL-6 governs the production of pro- and anti-inflammatory factors in airways during allergic inflammation.^[34]^ Eosinophils contribute to allergic inflammation partly by releasing a complex milieu of soluble mediators, and human bronchial fibroblasts respond by acquiring pro-inflammatory features, including IL-6 and IL-8 induction.^[35]^ Akira Iwamoto et al identified GAPDH as an IgE-inhibiting factor in strawberry extract, which suppressed IgE production both in vitro and in vivo. GAPDH also inhibited IgE in ovalbumin-stimulated human peripheral blood mononuclear cells while enhancing IgA, IgG, and IgM production.^[36]^ MMP9 has been shown to participate in mast cell activation, and inhibition of its activity or expression suppresses mast cell responses, offering novel therapeutic avenues for AR.^[37]^

Based on the preceding discussion, we demonstrate that QGBK’s therapeutic mechanism against AR comprises 3 integral components: Quercetin-mediated regulation of IgE release pathways directly correlating with clinically observed symptom alleviation; Kaempferol-driven suppression of mast cell activation accounting for the formulation’s rapid onset characteristics; Palmitic acid-facilitated lipid metabolism modulation providing the molecular basis for improved nasal mucosal remodeling, while molecular docking confirms stable binding between core targets (STAT3, IL-6, etc) and active constituents, with this targeting paradigm demonstrating extensibility to TCM compound research, although certain limitations merit consideration: active ingredient screening relied on database predictions requiring validation of actual blood-absorbed components, network analysis overlooked critical ADME parameters, and molecular docking outcomes await in vivo efficacy verification through animal models.

## 5. Conclusion

In summary, QGBK treats AR through a multi-component, multi-target, and multi-pathway mechanism. Quercetin, Kaempferol, Emodin, and Palmitic acid serve as the primary therapeutic components in QGBK, while STAT3, IL-6, GAPDH, and MMP9 are identified as key targets. This study substantiates the therapeutic efficacy of QGBK’s herbal components against AR, though their individual effects may not fully encapsulate the formula’s holistic complexity. Thus, future validation through integrated pharmacodynamics evaluation becomes imperative to advance both TCM compound research and clinical formulation development for AR therapeutics.

## Author contributions

**Conceptualization:** Shan Liu.

**Data curation:** Shan Liu.

**Investigation:** Jinglan Lin.

**Software:** Zhihao Huang.

**Visualization:** Lifen Chen.

**Writing – original draft:** Shan Liu.

**Writing – review & editing:** Yanwen Cai.
